# Spontaneous gas effusions: A rare complication of covid 19

**DOI:** 10.1016/j.amsu.2021.102508

**Published:** 2021-06-21

**Authors:** Rajae Ben Chaib, Mohammed Aabdi, Douaa Jakhjoukh, Manal Merbouh, Ikram Mekkaoui, Samia Berrichi, Ghizlane El Aidouni, Houssam Bkiyar, Brahim Housni

**Affiliations:** a Intensive Care Unit, Mohammed VI University Hospital, Faculty of Medicine and Pharmacy, Oujda, Morocco; b Simulation Center for Medical Formation, Morocco

**Keywords:** Pneumothorax, COVID-19, ARDS, ICU

## Abstract

Spontaneous gas effusion unrelated to assisted ventilation is a newly recognized complication of Severe acute respiratory syndrome coronavirus 2 (SARS-CoV-2). The objective of the present study was to examine the incidence, risk factors and the outcomes of Spontaneous gas effusions. 610 cases were analyzable, with 3 patients developing spontaneous gas effusion. This latter was associated with increased intubation and a trend towards death in one case. Drainage was required in two cases.

In conclusion, spontaneous gas effusions appeared to be a rare complication of severe acute respiratory syndrome. Further research is needed to investigate its pathogenesis.

## Highlights

• Several studies have demonstrated that Spontaneous gas effusion unrelated to assisted ventilation is a newly recognized complication of Severe acute respiratory syndrome coronavirus 2 (SARS-CoV-2).
• The objective of the present study was to examine the incidence, risk factors and the outcomes of Spontaneous gas effusions.
• Spontaneous gas effusions during COVID-19 should not be neglected as it is associated with severe outcomes.

## 1 Introduction

COVID-19 a new pandemic has become worldwide and manifests mainly by respiratory symptoms. Gas effusions may be spontaneous or secondary (traumatic or iatrogenic) [1]. Iatrogenic pneumothorax may occur during mechanical ventilation (invasive or non-invasive) or during intubation. During the Severe acute respiratory syndrome coronavirus 2 (SARS-CoV-2) pandemic spontaneous gas effusions were a rare complication. (see Fig. 6)

In this paper, we will describe3 cases of spontaneous gas effusions (two pneumothoraxes and one pneumoediastinum)with SARS COV 2 pneumopathy confirmed by polymerase chain reaction (PCR) and chest scan.

## 2 Case report

### 2.1 Case 1

We report the case of a 38-year-old patient with no medical history who was admitted to the intensive care unit for the management of respiratory distress.

His history reveals an influenza-like syndrome dating back to 08 days, a fever of 39.2 °C, arthralgia, myalgia associated with a dry cough, and anosmia with the notion of contact with a COVID-19 positive patient.

On his admission, clinical examination revealed a conscious patient, tachypneic at 24 cycles/min with pulse oxymetry at 74% on room air and 89% under 15 L/min of oxygen under high concentration mask, a heart rate of 82 beats per minute, and blood pressure at 122/65 mm Hg, with no evidence of right or left heart failure. Pulmonary auscultation found basithoracic crackles bilaterally.

Blood gas analysis revealed a pH7.3 of, a PaO 2 56 of mmHg, a PaCO 2 32 of mmHg.

Biological evaluation revealed an inflammatory syndrome, with hyperleukocytosis at18730 lymphopenia at 460 cells/μ L, C-reactive protein at 116 mg/L procalcitonin at 0.17 ng/ml, and ferritin at1502, fibrinogen level 7. 9 g/lthe rest was normal.

**Fig. 1:**
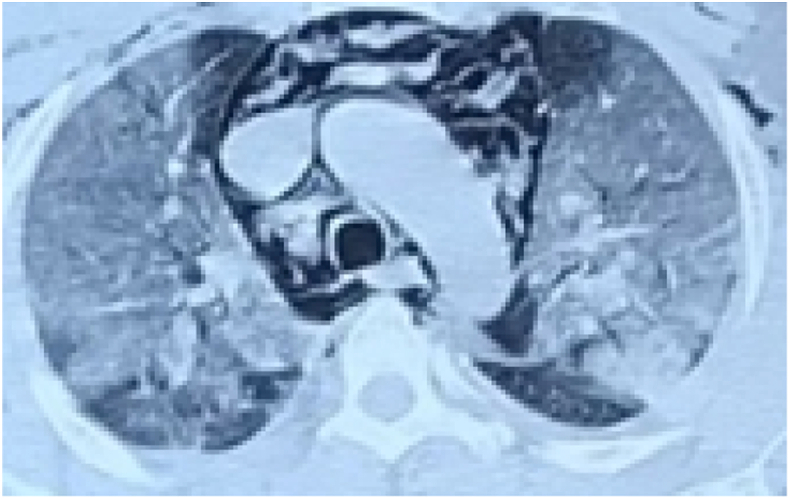
Chest CT scan in axial section of the parenchymal window showing ground glass images with an asymmetric peripheral arrangement under the pleural layer, characteristic of typical COVID-19 pneumonia.

The chest scan was showed bilateral, peripheral, and subpleural ground-glass opacities classified CORADS 5 with lung damage superior to 75% involvement associated with a pneumomediastinum and subcutaneous emphysema of the axillary, cervical, and anterior thoracic soft parts. (Fig. 1), polymerase chain reaction (PCR) for covid 19 infection was positive.

Therapeutic protocol associating azithromycin, dexamethasone 6mg/d kardegic 160 mg/d Vit C, Zinc and a curative anticoagulation Enoxaparin 100Ui/kg*2/d.a Bi-anti biotherapy based on triaxon 2g/d and ciprofloxacin 200 mg/12h was initiated.

The patient received a dose of tocilizumab 400 mg after 48 hours.

The evolution was favorable with the improvement of respiratory symptoms, and our patient was declared cured after 31 days and 2 negative PCRs on nasopharyngeal swabs at 24 hours intervals.

### 2.2 Case2

A 47-year-old patient with a pathological history of high blood pressure, diabetes, chronic renal failure with preserved diuresis, and a baseline creatinine of 25 mg/l, admitted to the intensive care unit for acute respiratory failure due to pneumonia caused by SARS CoV 2, evolving for 08 days.

The clinical examination on admission showed a conscious patient, hemodynamically stable with a blood pressure of 135/75 mmHg, a heart rate of 75 beats per minute, and pulse oxymetry of 70% on room air and 93% on 3L/min of oxygen.

The transthoracic echocardiography showed a left ventricle with preserved global systolic function with EF at 60%, normal filling pressures, absence of valvulopathies, with a non-dilated right ventricle of normal function, dry pericardium.

Blood gas analysis revealed a pH7.36 of, a PaO 2 50 of mmHg, a PaCO 2 32 of mmHg.

The biological evaluation revealed an inflammatory syndrome, with hyperleukocytosis to12080 lymphopenia to 300 cells/μ L, C-reactive protein to 189 mg/L and procalcitonin to 2.21 ng/ml, ferritin to1387, fibrinogen level 6.2g/l hemoglobin level to 6 g/dL, renal failure with urea to 1.59 mmol/L and creatinine to 87.39 μ mol/L.

**Fig. 2:**
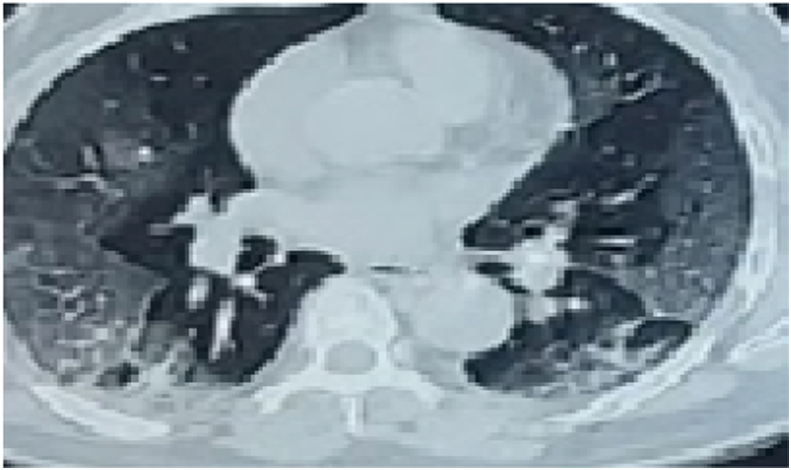
Chest scan in axial section of the parenchymal window showing bilateral, peripheral, and subpleural opacities in ground glass, and basal condensations.

The chest scan was performed showed bilateral, peripheral, and subpleural opacities in ground glass classified CORADS 5 with 75% of lung damage and basal condensations (Fig. 2).

The evolution was marked 5 days later by the aggravation of respiratory signs requiring oxygen therapy with a high concentration face mask up to 10 L/min.

**Fig. 3:**
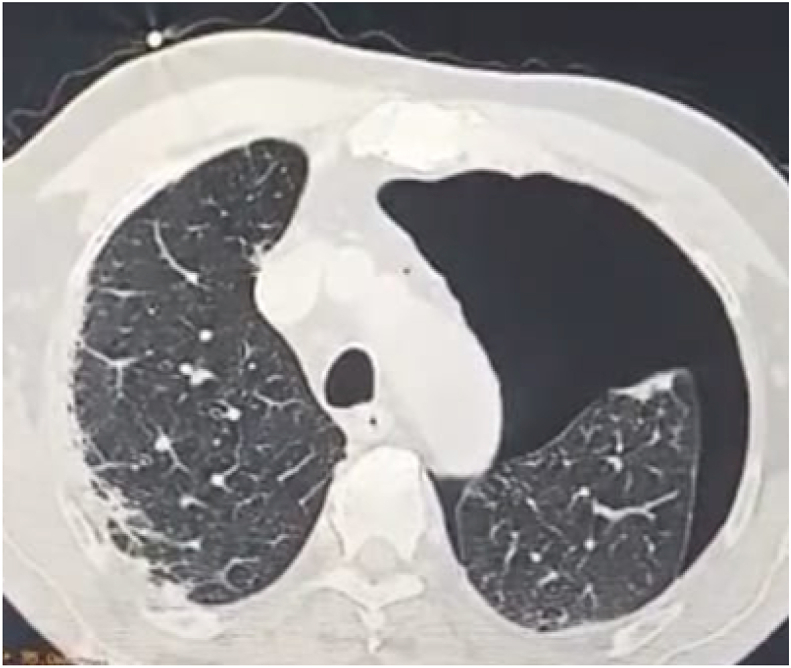
Chest scan of the parenchymal window revealed a left spontaneous pneumothorax.

A second chest scan of the thorax was performed on the 10th day of hospitalization revealing a left spontaneous pneumothorax (Fig. 3).

**Fig. 4:**
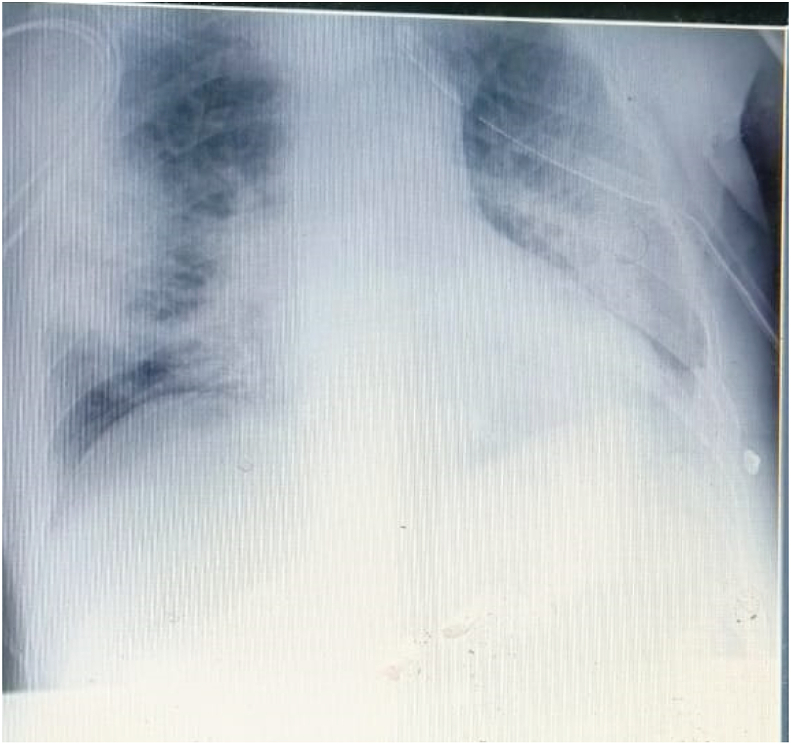
Chest -X-Ray realized after pneumothorax drainage.

The effusion was drained and the evolution was favorable with discharge after 21 days of hospitalization (Fig. 4).

### 2.3 Case 3

A 66-year-old male, with a history of diabetes, was admitted to the intensive care unit for management of acute respiratory failure with chest pain on the right side of the chest that appeared suddenly 3 days earlier with dry cough.

The patient had no history of chest trauma and no fever or other systemic symptoms. On examination of his vital signs, the temperature was 37° and blood pressure (135/75 mm Hg), and a heart rate of 77 beats per minute. He had a respiratory rate of 18 cycles per minute, oxygen saturation of 66% on room air, and 84% on 15. His body mass index was 22 kg/m2. Blood test results showed lymphopenia at 300/μ L, white blood cells at 11,610, CRP 0.48mg/L.ferritin level at2797, fibrinogen at 8.1 g/l.normal hemoglobin level at 13g/dl

**Fig. 5:**
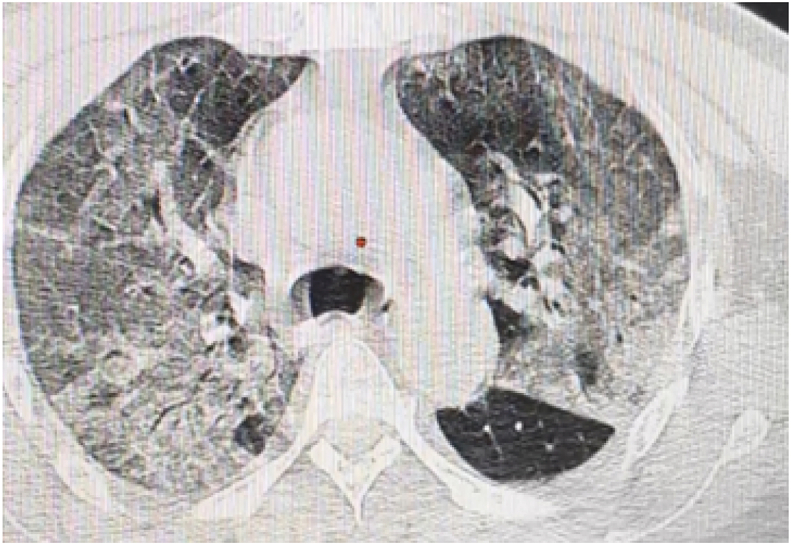
Chest scan in axial section of the parenchymal window showing bilateral ground-glass opacities with 80% severe parenchymal damage.

A thoracic chest scan revealed bilateral ground-glass opacities with 80% severe parenchymal damage (Fig. 5).

**Fig. 6:**
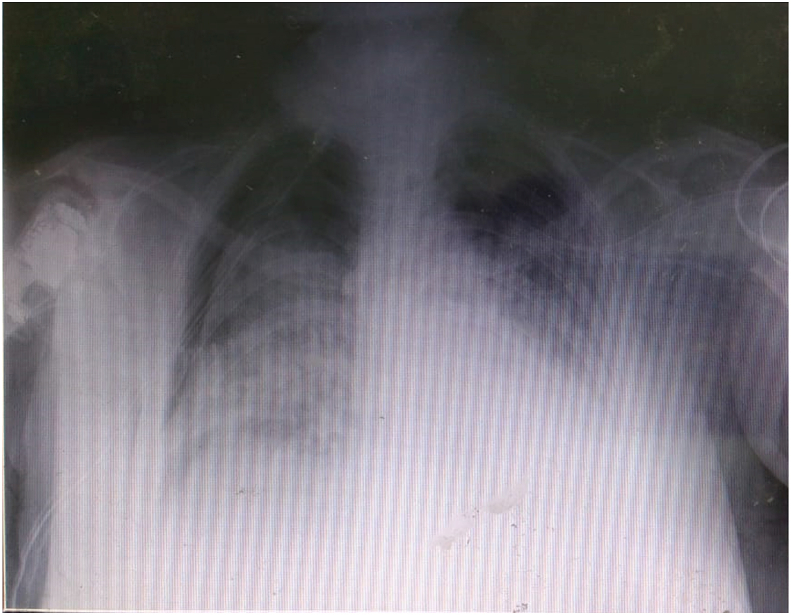
Chest -X-Ray showed a right pneumothorax.

The evolution was marked 07 days later by the aggravation of respiratory signs requiring oxygen therapy by a high flow nasal probe (optiflow) with a flow rate of 40/min inspired oxygen fraction 90% temperature 34.

Thoracic radiography was carried out on the 8th day of hospitalization and showed a right pneumothorax which required thoracic drainage.

Due to the severe hypoxemia, the patient was intubated after 10 days of hospitalization with a bad evolution and death.

**Table:** 

cases	Sex-age-history	Clinical signs	CT Imaging	Evolution
Case 1	Male 38 years old 0 history	Influenza-like syndrome (08 days) + fever+cough-arthralgia-myalgia-anosmia	J8 bilateral, peripheral and subpleural ground-glass opacities, with upper 75% involvement associated with a pneumomediastinum of great abundance associated with subcutaneous emphysema of the axillary, cervical and anterior thoracic soft parts	Favorable under high concentration oxygen mask Exit after 31 days
Case 2	Male 47 years old History: HTA-Chronic respiratory failure-diabetes-diabetic retinopathy +diabetic neuropathy	Dyspnea+cough going back 8 days	Chest CT (d8 symptoms): bilateral peripheral and subpleural ground-glass opacities, classified as CORADS 5 with greater than 75% involvement, foci of basal condensations CT scan (j13): revealing a right spontaneous pneumothorax.	Drainage of the pneumothorax Favourable evolution with disappearance of the PS and discharge after 21 days of hospitalization
Case3	Male 66 years old diabetes	Cough-dyspnea Chest pain dating back 3 days	CT scan on day 10: bilateral ground glass opacities with 80% severe parenchymal involvement Chest X-ray on day 8 of hospitalization: right pneumothorax	D10 of hospitalization intubation and mechanical ventilation death

## 3 Discussion

A pneumothorax can develop spontaneously, either as a primary spontaneous pneumothorax or as a secondary spontaneous pneumothorax due to an underlying lung disorder. It may be caused by chest trauma, or it may be iatrogenic. Risk factors for spontaneous pneumothorax include smoking and cannabis, male gender, tall height, thin body habitus, chronic obstructive pulmonary disease, alpha-1 antitrypsin deficiency, cystic fibrosis, other cystic lung disorders, malignancy, lung infections, or architectural abnormalities such as Marfan syndrome, Ehlers-Danlos syndrome, or homocystinuria [2,3].

Our research found several cases of spontaneous pneumothorax, the majority of patients were men without a history of respiratory pathology, and the usual risk factors of COVID-19 (age > 60 years, diabetes, hypertension cardiovascular diseases, etc.) were reported in some patients. This rare complication can also be observed in women, as in the study by L. Gorospe and al [4] (2 women and 2 men). And it can affect even young people with no previous history as is the case in a case report from Turkey [5] where a 24-year-old non-smoking male presented with COVID-19 pneumonia and a large left pneumothorax.

In our study spontaneous pneumothorax appeared secondarily on CT scan in two patients (d10-j13) after the onset of clinical symptoms of COVID-19) with early-onset on chest CT scan at d8 of pneumomediastinum symptomatology in one patient; all patients had initial CT scan on admission. All three patients had extensive bilateral severe pneumonia, according. and all 3 patients had repetitive intense episodes of dry cough, these latter are known to produce a sudden increase in distal airway pressure, causing an alveolar rupture and secondary gas leakage to the peribronchovascular pulmonary interstitium, from which air can dissect proximally, eventually reaching the mediastinum. This phenomenon, called the “Macklin effect”, has been implicated as the cause of pneumomediastinum that occurs in certain closed-chest lesions, asthma attacks and Valsalva maneuvers [6]. Diagnosis is based on chest X-ray and/or especially chest CT scan. The frontal chest X-ray is normal in 50% of cases. It may show an areal border along the left edge of the cardiac silhouette, subcutaneous emphysema (subcutaneous hyperclarity of the cervical region), and Minnigerode's sign (presence of air in the paraesophageal region of the neck), which is an early sign of pneumomediastinum. If the front view is normal, the profile view looks for retrosternal clarity and clear lines bordering the aortic arch and left pulmonary artery. The thoracic CT scan shows air dissection of all the anatomical structures of the mediastinum and neck, may show an associated pneumo-pericardium, and allows differentiating pneumomediastinum from anterior pneumothorax. During spontaneous pneumothorax, the CT scan shows the integrity of the tracheobronchial tree and the digestive structures. The natural evolution of spontaneous pneumothorax is towards recovery, but in some cases, the pneumothorax can be associated with a tendency towards intubation and mortality especially in severe pneumonia (our 3rd case). No specific therapy seems to have proven its effectiveness. Treatment is symptomatic (bed rest, analgesics and possibly nasal oxygen therapy). Recurrences appear to be rare. Complications are exceptional [7].

## 4 Conclusion

Spontaneous gas effusions revealing SARS-CoV-2 pulmonary infection is a rare entity. It is probably secondary to a diffuse alveolar lesion causing an alveolar rupture and an air leak. The treatment in most cases is conservative but sometimes it can be associated with a tendency towards intubation and increased mortality.

The work has been reported in line with the CARE 2018 criteria [8].

## Conflicts of interest

None.

## Sources of funding

None.

## Ethical approval

The ethical committee approval was not required give the article type (case report).However, the written consent to publish the clinical data of the patients was given and is available to check by the handling editor if needed.

## Consent

Obtained.

## Author contributions

Rajae, Ben Chaib Rajae: Study concept, Data collection, Data analysis, Writing the paper.Mohammed, Aabdi Mohammed: Study concept, Data analysis, Writing the paper Jakhjoukh Douaa: Data collection Berrichi Samia: Contributor Houssam Bkiyar: Supervision and data validation Brahim Housni: Supervision and data validation.

## Registration of research studies

This is not an original research project involving human participants in an interventional or an observational study but a case report. This registration is was not required.

## Guarantor

Dr Ben Chaib Rajae

Dr Aabdi Mohammed
